# Naoqing formula alleviates acute ischaemic stroke-induced ferroptosis via activating *Nrf2*/xCT/GPX4 pathway

**DOI:** 10.3389/fphar.2024.1525456

**Published:** 2024-12-17

**Authors:** Yujun Ye, Xuexin Xie, Yiming Bi, Qing Liu, Xuliang Weng, Lingling Qiu, He Zhao, Shangyan Hei, Ling Yang, Chengyin Wang, Weifeng Zhu, Ting Zeng

**Affiliations:** ^1^ The Affiliated Traditional Chinese Medicine Hospital, Guangzhou Medical University, Guangzhou, Guangdong, China; ^2^ School of Combine Traditional Chinese and Western Medicine, Guangzhou Medical University, Guangzhou, Guangdong, China; ^3^ Institute of Integration of Traditional and Western Medicine of Guangzhou Medical University, Guangzhou Medical University, Guangzhou, Guangdong, China

**Keywords:** acute ischaemic stroke (AIS), Naoqing formula (NQ), *Nrf2*, ferroptosis, oxidative stress

## Abstract

**Backgrounds:**

Ferroptosis is a form of regulated cell death. The accumulation of iron in the brain is linked to trigger ferroptosis after an ischaemic stroke (IS). Naoqing formula (NQ) is a traditional Chinese medicine metabolites with the clinical function of activating blood circulation, which is applied to treat IS clinically in China.

**Methods:**

Mice and SH-SY5Y cells were utilized to investigate the protective effects and the underlying mechanism of NQ against middle cerebral artery occlusion (MCAO) induced acute ischaemic stroke (AIS) and neuronal cellular ferroptosis caused by ferroptosis inducer Erastin *in vitro* and *in vivo*. Utilizing molecular biological techniques, transcriptomics, and proteomics analyses, the role of NQ in *Nrf2* regulation and ferroptosis was evaluated through the pharmacologic inhibition of *Nrf2*.

**Results:**

NQ attenuated AIS-induced neuronal damage and cerebral infarction by increasing cortical blood flow (CBF). Transcriptomics and proteomics analyses revealed that NQ might regulate lipid and iron metabolism through *Nrf2* pathway. Additionally, NQ can protect AIS from ferroptosis by reducing oxidative stress and iron overload. Meanwhile, *Nrf2*, solute carrier family 7 member 11 (SLC7A11; also known as xCT) and glutathione peroxidase 4 (GPX4) were upregulated in NQ-treated AIS mice. Consistent with the results *in vivo*, NQ led to ferroptosis resistance upon exposure to a ferroptosis-inducing compound through activation of *Nrf2*/xCT/GPX4 pathway *in vitro.* Notably, *in vivo* inhibition of *Nrf2* expression by ML385 aggravated the ferroptotic events and weakened the neuroprotective effect of NQ as well as subsequently reduced the expression of xCT and GPX4.

**Conclusion:**

This study demonstrated that NQ protected against AIS via suppression of ferroptosis and oxidative stress, which were largely dependent on the upregulation of *Nrf2* pathway.

## Graphical Abstract

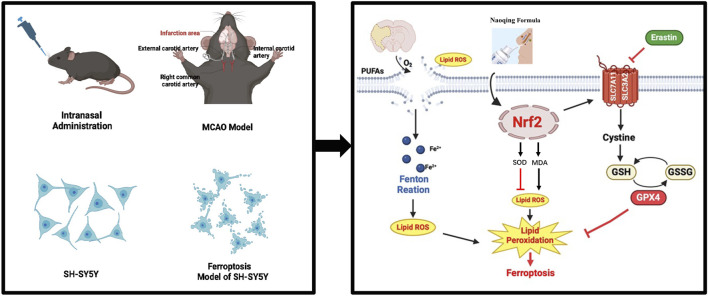

## 1 Introduction

Acute ischaemic stroke (AIS) is a common disease worldwide with high mortality and disability rates (Campbell et al., 2019). In the pathogenesis of acute ischaemic stroke, multiple mechanisms of cell death occur, including necrosis, apoptosis and autophagy (Qin et al., 2022; Wu et al., 2018). In 2012, Dixon et al. initially introduced the concept of ferroptosis, a type of programmed cell death that is reliant on iron and is distinctly characterized by lipid peroxidation (Dixon et al., 2012). This process is characterized by the lethal levels of lipid hydroperoxides dependent on iron accumulation within cells (Dodson et al., 2019). Ferroptosis directly or indirectly inhibits glutathione peroxidase 4 (GPX4) and the cystine/glutamate transporter system xc-/xCT (SLC7A11), leading to intracellular damage to antioxidant systems and mitochondrial accumulation of reactive oxygen species (ROS), ultimately causing cellular dysfunction (Dodson et al., 2019; Imai et al., 2017). Recent investigations have emphasized the significant role of ferroptosis in a diverse array of neurological conditions, spanning strokes, degenerative pathologies, neurotrauma, and malignancies (Quaegebeur et al., 2016). This revelation sheds new light on the potential of targeting ferroptosis for therapeutic interventions in these disorders. Hence, regulating neuronal death through ferroptosis could be a plausible approach for treating AIS. Studies indicate that cells significantly enhance *Nrf2* expression in the acute phase of stroke (Michaličková et al., 2020). *Nrf2*, a pivotal regulatory factor, exerts inherent neuroprotective effects and is involved in is involved in mediating cellular antioxidant response, cellular protection and anti-inflammatory genes (Kerins and Ooi, 2018). Under normal circumstances, *Nrf2* levels remain low due to Keap1-mediated proteasome degradation. However, during oxidative stress, *Nrf2* detaches from the anchoring protein Keap1, translocates to the nucleus and modulates the expression of various downstream antioxidant stress proteins, detoxifying enzymes and transporter genes, thereby contributing to physiological and pathological responses to antioxidant stress (Kerins and Ooi, 2018; Suzuki et al., 2013). Reports indicate that *Nrf2* exhibits resistance to ferroptosis by modulating the expression of SLC7A11, GSH, and GPX4, thereby playing a protective role in cellular defense mechanisms (Ishii et al., 2000; Osburn et al., 2006). The *Nrf2*/GPX4 pathway is an antioxidant pathway related to ferroptosis. Studies have shown that activating this pathway can reduce iron accumulation, thereby alleviating the neural damage caused by ferroptosis after brain injury. GSH can donate an electron to GPX4, facilitate the elimination of harmful lipid peroxides. GPX4 is regarded as a GSH-dependent enzyme, which influences GSH expression levels in cells (Kerins and Ooi, 2018). The SLC7A11 gene belongs to the solute transport family and is responsible for encoding a crucial cystine/glutamate xCT transporter. This transporter plays a significant role in regulating the process of “iron overload-ferroptosis” (Lang et al., 2019). After SLC7A11 inhibition, GSH synthesis ceases, GPX4 cannot eliminate lipid peroxide, and iron-induced cell death begins (Yang et al., 2014). Hence, *Nrf2*, as a crucial element in maintaining iron homeostasis and managing oxidative stress, plays an indispensable part in controlling ferroptosis. Its significance as a potential target for addressing ferroptosis triggered by AIS cannot be understated. This study provides a valuable basis and a novel therapeutic approach for the treatment of IS.

In China, natural botanicals are extensively employed for the management of IS, exhibiting diverse therapeutic benefits through the modulation of numerous targets and signaling pathways related to IS (Li et al., 2022). Musk (artificial breeding) and six traditional Chinese botanical drugs make up NQ, *Dryobalanops aromatica* C.F.Gaertn. [Dipterocarpaceae; *Dipterocarpus camphora*], *Ligusticum striatum* DC. [*Ligusticum* chuanxionghort], *Acorus tatarinowii* Schott [Acori Tatarinowii Rhizoma],*Panax notoginseng* (Burkill) F.H.Chen [Araliaceae; *Panax notoginseng*]and *Mentha canadensis* L. [Lamiaceae; dementholised mint oil], their names have been checked through MPNS (http://mpns.kew.org). Musk is the dried secretion of the scent sac of mature male musk deer bred artificially. The theory of communication between the nose and the brain in TCM was first described in “The Yellow Emperor’s Canon of Internal Medicine”. Transnasal administration of drugs for the treatment of encephalopathy, in the selection of drugs is more focused on the selection of pungent and aromatic travelling and stimulating drugs. Musk was first recorded in the “The Shennong Herbal Classics”. It has revitalising, blood-activating and swelling-dissolving properties and is used to treat strokes (Chinese Pharmacopoeia Commission, 2020; Shennong’s Herbal Classic, 2007). Muscone is the main active ingredient in musk, and studies have shown that muscone can alleviate neuronal damage by reducing apoptosis in acute IS (Sun et al., 2024). In clinical applications, musk frequently serves as the monarch of complex formulations designed for the treatment of IS, encompassing formulations like Angong Niuhuang pills (Liu et al., 2019) and Xingnaojing injection (Tian et al., 2021). *Dryobalanops aromatica* C.F.Gaertn. is classified as a “Courier”herb, which refers to the delivery of other active herbs to target organs, especially in the upper parts of the body, such as the brain. Therefore, TCM suggests that *Dryobalanops aromatica* C.F.Gaertn. is mainly used to treat various central nervous system (CNS) disorders such as dementia, stroke, IS and coma (Chen et al., 2019; Kulkarni et al., 2021; Yang et al., 2009). According to research, borneol displays a protective capability against neuronal damage caused by extramitochondrial oxygen-glucose deprivation/reperfusion (OGD/R). Notably, it reverses the deleterious effects of reperfusion, mitigates intracellular ROS production, and prevents the dissipation of mitochondrial membrane potential (Liu et al., 2011), and to improve cerebral infarct size and neurobehavioral scores (Chang et al., 2017). A randomized, double-blinded, comparative, multicenter Phase III clinical trial has successfully demonstrated the effectiveness of an injection containing the active component (+)-borneol in treating patients suffering from acute IS (Xu et al., 2021). *Ligusticum striatum* DC. is first recorded in “The Shennong Herbal Classics”. It is an ascending herb that specialises in the treatment of all diseases of the brain and has the effect of invigorating the blood and promoting the circulation of “Qi” (Shennong’s Herbal Classic, 2007). *Ligusticum striatum* DC. and *Dryobalanops aromatica* C.F.Gaertn. are effective in ischemia/reperfusion (I/R) injury and are widely used in the clinic (Wang et al., 2024). Studies have shown that the main components of *Acorus tatarinowii* Schott have positive effects on transplanted neural progenitor cells in a mouse model of IS (Lee et al., 2018). The combination of *Ligusticum striatum* DC. and *Acorus tatarinowii* Schott are also common components of clinical treatments for IS, as exemplified by the Huangxiong formula (Zhao et al., 2023). In clinical practice, *Panax notoginseng* (Burkill) F.H.Chen is employed primarily to enhance blood circulation and facilitate the resolution of bruising, as Xueshuantong (Kjaergard et al., 2001). It has been demonstrated that *Panax notoginseng* (Burkill) F.H.Chen can prevent apoptosis induced by OGD/R models (Chen et al., 2021). A major component of *Mentha canadensis* L. has been found to reduce the size of infarcts and improve sensorimotor deficits in mice with CIRI (Huang S. S et al., 2022). In Chinese medicine, stroke is attributed to a deficiency of the internal organs, an impairment of the flow of “Qi” and blood, and an obstruction of the phlegm and blood stasis, which collectively result in the clouding of the clear orifices and the subsequent onset of the disease. Analysing the TCM etiology and pathogenesis of stroke and establishing the connection between ferroptosis and stroke’s etiology and pathogenesis can facilitate the precise intervention of traditional Chinese medicine in the post-stroke ferroptosis process. Both ROS and cerebral iron deposition observed in early stroke stages indicate initial nerve cell injury due to stasis and stagnation of collaterals (Lu et al., 2020). In the management of CIRI, the integration of six Chinese herbal medicines embodies the therapeutic tenets of nourishing ‘Qi’ (Yi-Qi) and promoting blood flow to alleviate blood stagnation (Huo-Xue-Hua-Yu), thereby addressing the underlying pathological mechanisms. Notably, NQ is used for nasal drug delivery, offering high bioavailability and bypassing the gastrointestinal liver’s first-pass effect, effectively engaging its role across the BBB (Mittal et al., 2014). Nevertheless, the molecular mechanisms by which NQ affects AIS remain to be fully elucidated.

Drawing from these discoveries, we hypothesize that NQ alleviates AIS through the modulation of *Nrf2*-mediated ferroptosis. This research endeavors to investigate the neuroprotective efficacy and underlying protective mechanisms of NQ in the context of AIS.

## 2 Materials and method

### 2.1 Preparation of NQ formula

NQ was prepared by Guangdong Pharmaceutical University, and the botanical drugs in NQ were obtained from the Affiliated Traditional Chinese Medicine Hospital of Guangzhou Medical University, Musk was provided by Foshan Dezhong Pharmaceutical. Naoqing formula consists of drugs: *Ligusticum striatum* DC. [*Ligusticum* chuanxiong hort], *Panax notoginseng* (Burkill) F.H.Chen [Araliaceae; *Panax notoginseng*]and *Mentha canadensis* L. [Lamiaceae; dementholised mint oil], Musk, *Dryobalanops aromatica* C.F.Gaertn. [Dipterocarpaceae; *Dipterocarpus camphora*], *Acorus tatarinowii* Schott [Acori Tatarinowii Rhizoma]. *Mentha canadensis* L, at a ratio of 29:29:3.3:3.3:1:1 (Table 1). Oil and *Acorus tatarinowii* Schott Oil were extracted with triple-distilled water, *Panax notoginseng* (Burkill) F.H.Chen and *Ligusticum striatum* DC. were extracted with ethanol (separated and adsorbed with a macroporous resin), Musk and *Dryobalanops aromatica* C.F. Gaertn. were dissolved in ethanol. The drugs used in the *in vitro* cell assay were freeze-dried drugs, and the freeze-drying conditions were: refrigeration at 40°C, freezing at −20°C for 2 h, freezing at −10°C for 16 h, drying at 20°C for 36 h, and secondary drying at 35°C for 36 h. Weigh the freeze-dried NQ, dissolve it with DMSO to a final concentration of 10 mg/mL, centrifuge at 15000 rpm for 10 min, and filter the supernatant with a 0.22 μL filter membrane for later use.

**TABLE 1:** The components of NQ.

Chinese name	Full name	Part used	Weight(g)
She Xiang	Musk	Dried secretion of the scent sac	5 g
Bing Pian	*Dryobalanops aromatica* C.F.Gaertn	Crystal	5 g
San Qi	*Panax notoginseng* (Burkill) F.H.Chen Extract	Root	43.5 g
Chuan Xiong	*Dryobalanops aromatica* C.F.Gaertn.Extract	Rhizome	43.5 g
Bo He	*Mentha canadensis* L. Oil	Leave	1.5 g
Shi Changpu	*Acori Tatarinowii* Schott Oil	Rhizome	1.5 g

### 2.2 Analysis of NQ by UPLC-QE-MS and GC-MS

Batch consistency was monitored by UPLC-QE-MS and GC-MS analysis of NQ major components and GC-MS quantitative analysis of muscone. Determination of components in NQ was performed on an UHPLC system (Vanquish, Thermo Fisher Scientific) with a Waters UPLC BEH C18 column (1.7 μm 2.1*100 mm). The sample injection volume was set at 5 μL. The flow rate was set at 0.5 mL/min. The mobile phase consisted of 0.1% formic acid in water (A) and 0.1% formic acid in acetonitrile (B). The multi-step linear elution gradient program was as follows: 0–11 min, 85%–25% A; 11–12 min, 25%–2% A; 12–14 min, 2%–2% A; 14–14.1 min, 2%–85% A; 14.1–15 min, 85%–85% A; 15–16 min, 85%–85% A. The main volatiles in NQ were detected using GC-MS. In SPME cycle of PAL rail system. Incubate temperature is 30°C; Preheat time in 15 min; Incubate time in 30 min; Desorption time is 4 min.GC-MS analysis was performed using an Agilent 7890 gas chromatograph system coupled with a 5977 B mass spectrometer. The system utilized a DB-Wax. Injected in Splitless Mode. Helium was used as the carrier gas, the front inlet purge flow was 3 mL min^−1^, and the gas flow rate through the column was 1 mL min^−1^. The initial temperature was kept at 40°C for 4 min, then raised to 245°C at a rate of 5 °C min^−1^, kept for 5 min. The injection, transfer line, ion source and quad temperatures were 250, 250, 230°C and 150°C, respectively. The energy was −70 eV in electron impact mode. The mass spectrometry data were acquired in scan mode with the m/z range of 20–400, solvent delay of 2.37 min. The determination of each metabolite in NQ, quality control of muscone, fingerprinting methods for drugs were listed in Supplementary Material S1.

### 2.3 Animals

Male C57BL/6 mice weighing 21–25 g were housed in certified pathogen-free conditions, maintaining a light/dark cycle (25°C ± 2°C and 60% – 70% relative humidity). Adequate food and drink were provided. The Animal Laboratory of Guangzhou Topbioteeh Co. Ltd.’s Ethics Committee approved all pertinent operational research, which was overseen by the Animal Experiment Center (licence number: SYXK(YUE)2023-0315).

### 2.4 MCAO model

The mice were anaesthetised with 2% pentobarbital sodium and placed in a supine position. Sequential dissection of the right common carotid artery (CCA), external carotid artery (ECA) and internal carotid artery (ICA) was performed by carefully dissecting the arteries through a median incision in the mice’s anterior skin. The CCA and ECA were ligated step by step while simultaneously clamping the ICA with an arterial clamp. A silicone nylon monofilament was introduced through the CCA into the middle cerebral artery and secured temporarily. After 1 hour of ischaemia, the monofilament was removed, and the blood vessels were tied at the incision site. Surgical sutures were employed to close the neck wound. After 24 h of reperfusion, further experiments were conducted. Sham-operated mice underwent identical procedures without monofilament insertion. The cerebral cortex blood flux (CBF) was assessed using laser speckle contrast imaging/LSCI (RWD Life Science, China) before, during and after the operation to confirm MCA obstruction and blood flow changes.

### 2.5 Animal treatment groups

In this study, the clinical dose of NQ administered was 14.26 mg/kg/day, equating to 1 mL of nasal spray per day. To translate this dose to mice, we employed the formula: equivalent dose in mice (mg/kg) = 9.01 × human dose (mg/kg), resulting in a dose of 128.34 mg/kg. Consequently, we adjusted the NQ doses for mice to 130 mg/kg/day (low), 260 mg/kg/day (medium), and 520 mg/kg/day (high). The mice were randomly assigned to seven groups: Sham operated, Sham + 520 mg/kg NQ, MCAO, 130 mg/kg NQ, 260 mg/kg NQ, 520 mg/kg NQ, 10 mg/kg Edaravone (EDA) (positive control drug), *Nrf2* inhibitor ML385 (30 mg/kg), and *Nrf2* inhibitor ML385 (30 mg/kg) combined with 520 mg/kg NQ. ML385 and was dissolved in a solution comprising 5% DMSO, 40% PEG300, 5% Tween80, and 50% ddH_2_O. NQ was administered via nasal instillation, while ML385 and EDA were injected intraperitoneally. A total of seven administrations were given over 6 days before ischaemia/reperfusion, with the final dose administered 1 hour before the surgical procedure.

### 2.6 Cell culture and establishment of the ferroptosis model

SH-SY5Y neuroblastoma cells were obtained from Pricella (CL-0208, China) and cultured in F12 medium (Gibco, Gaithersburg, United States) containing 15% FBS (ExcellBio, FSD500) and 1% penicillin-streptomycin (Gibco Gaithersburg, United States) at 37°C in a humidified 5% CO_2_ environment. The ferroptosis metamorphosis model was induced using Erastin (Selleck).

### 2.7 Statistical analysis

For the remaining experiments in this study, GraphPad Prism 9.0 software was utilized for statistical analyses. The data presented feature a minimum of n = 6 per group, expressed as the mean with the standard error of the mean (SEM). Each experiment was replicated at least three times, ensuring the inclusion of at least three independent individuals per group. Additionally, a minimum of three independent replicate experiments, encompassing both Western blot (WB) and staining experiments, were conducted. For comparing means, an unpaired two-tailed t-test was employed, while a one-way ANOVA was used to assess differences in experiments involving multiple groups. Statistical significance was determined when p-values were less than 0.05, annotated as *p < 0.05, **p < 0.01, ***p < 0.001, and ****p < 0.0001. The sample size for each experimental condition is indicated using symbols in the accompanying figures. The remaining experimental methodology was described in Supplementary Material S2.

## 3 Results

### 3.1 Naoqing formula (NQ) attenuates AIS *in vivo*

**FIGURE 1:**
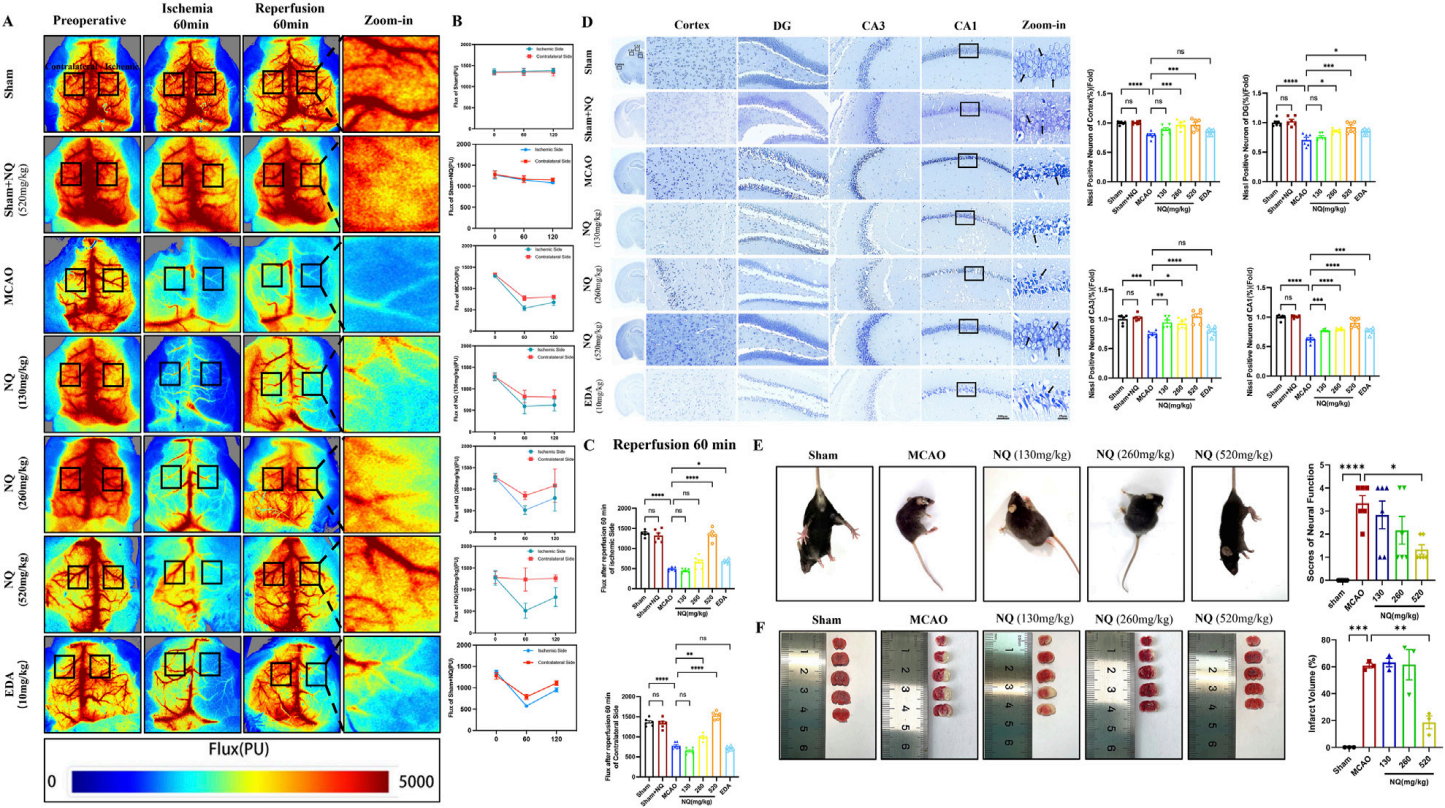
Naoqing formula (NQ) attenuates AIS *in vivo* **(A)** Representative raw data from laser speckle contrast imaging (LSCI) with defined regions of interest (ROIs) on the ischaemic and contralateral sides. **(B)** Flux values of ROIs in AIS mice preoperatively, 60 min of ischaemia and 60 min of reperfusion (n = 6/group). **(C)** Differences in flux changes between the ischaemic and contralateral sides at 60 min of reperfusion in the sham, sham + NQ,MCAO, NQ, EDA dose groups (n = 6/group). **(D)** Nissl-stained sections of the cerebral cortex, hippocampal DG, CA1 and CA3 regions from representative mice within each group (scale bar = 50 μm/20 μm, n = 6/group). **(E)** Neurobehavioral scores of mice were recorded at the time of postoperative awakening according to the Zea-Longa neurobehavioral score in the sham, MCAO and NQ dose groups (n = 6/group). **(F)** Brain sections of the sham, MCAO and NQ dose groups stained with TTC to show ischaemic cerebral infarction volume (n = 3/group). Bars represent mean ± SEM, statistical analysis was performed using one-way ANOVA with Tukey’s *post hoc* test. *p < 0.05, **p < 0.01, ***p < 0.001 and ****p < 0.0001.

The chemical metabolites in NQ samples were analyzed using UHPLC-QE-MS and GC-MS, and the negative and positive ion chromatograms were shown in Supplementary Material S1. The main active metabolites in NQ were muscone, cholesterol, endo-borneol, isoborneol, ligustilide, beta-asarone, ginsenoside rg5 and (−)-menthone. The quality control of muscone content in NQ were shown in Supplementary Material S1. To verify the role of NQ in AIS, a MCAO-induced AIS model was used, and CBF was assessed in AIS mice. There was no significant change in CBF before surgery, after 60 min of ischemia and after 60 min of reperfusion in the Sham group and the Sham + NQ group, while the CBF of the MCAO group and the NQ group decreased at 60 min of ischemia, and the CBF recovered at 60 min of reperfusion but did not return to normal levels (Figures 1A, B), indicating that NQ did not cause adverse reactions to the blood flow of mice, and MCAO could successfully block the blood flow and cause AIS. Compared with the sham group, the CBF of the ischemic side and the contralateral side of the MCAO group decreased after 60 min of reperfusion (Figure 1C). Pretreatment with 520 mg/kg NQ reduced the degree of bilateral CBF infarction at 60 min of reperfusion compared with the MCAO group (Figure 1C), and EDA increased the CBF blood flow of the ischemic side at 60 min of reperfusion compared with the MCAO group, but the improvement was not as good as that of 520 mg/kg NQ (Figure 1C). Nissl staining showed that after MCAO treatment, a large number of neurons in the cerebral cortex and hippocampus (CA1, CA3 and DG) were disordered, the cells were dissolved, and the nuclei were shrunken; after pretreatment with 520 mg/kg NQ, the neurons were neatly arranged, the nuclei were centered, the degree of necrosis was reduced, the Nissl vesicles were well preserved, and the cell structure was better than that of the MCAO group. EDA protected the integrity of some Nissl bodies, but the protective effect was not as good as that of 520 mg/kg NQ (Figure 1D). HE staining also showed that in MCAO mice, the cells in the cerebral cortex and hippocampus were lysed and the nuclei were shrunken; in mice treated with 520 mg/kg NQ, the cells were arranged neatly, the nuclei were centered, and the degree of necrosis was reduced (Supplementary Material S3). We recorded neurological scores according to the Zea-longa neurobehavioral scoring criteria at the time of postoperative awakening in AIS mice. Behavioral scores were reduced in the NQ cohort compared to the MCAO group, suggesting that NQ promotes the recovery of neurological function in AIS mice (Figure 1E). Furthermore, the area of cerebral infarction induced by MCAO was significantly reduced following NQ pre-treatment, as visualised using TTC staining (Figure 1F). The results confirmer that NQ can effectively improve CBF, reduce cerebral infarction volume, and prevent and treat brain tissue damage and neurological function damage caused by AIS.

### 3.2 Based on transcriptomic and proteomic analysis, NFE2L2 (*Nrf2*) is a potential target for NQ to alleviate AIS

**FIGURE 2:**
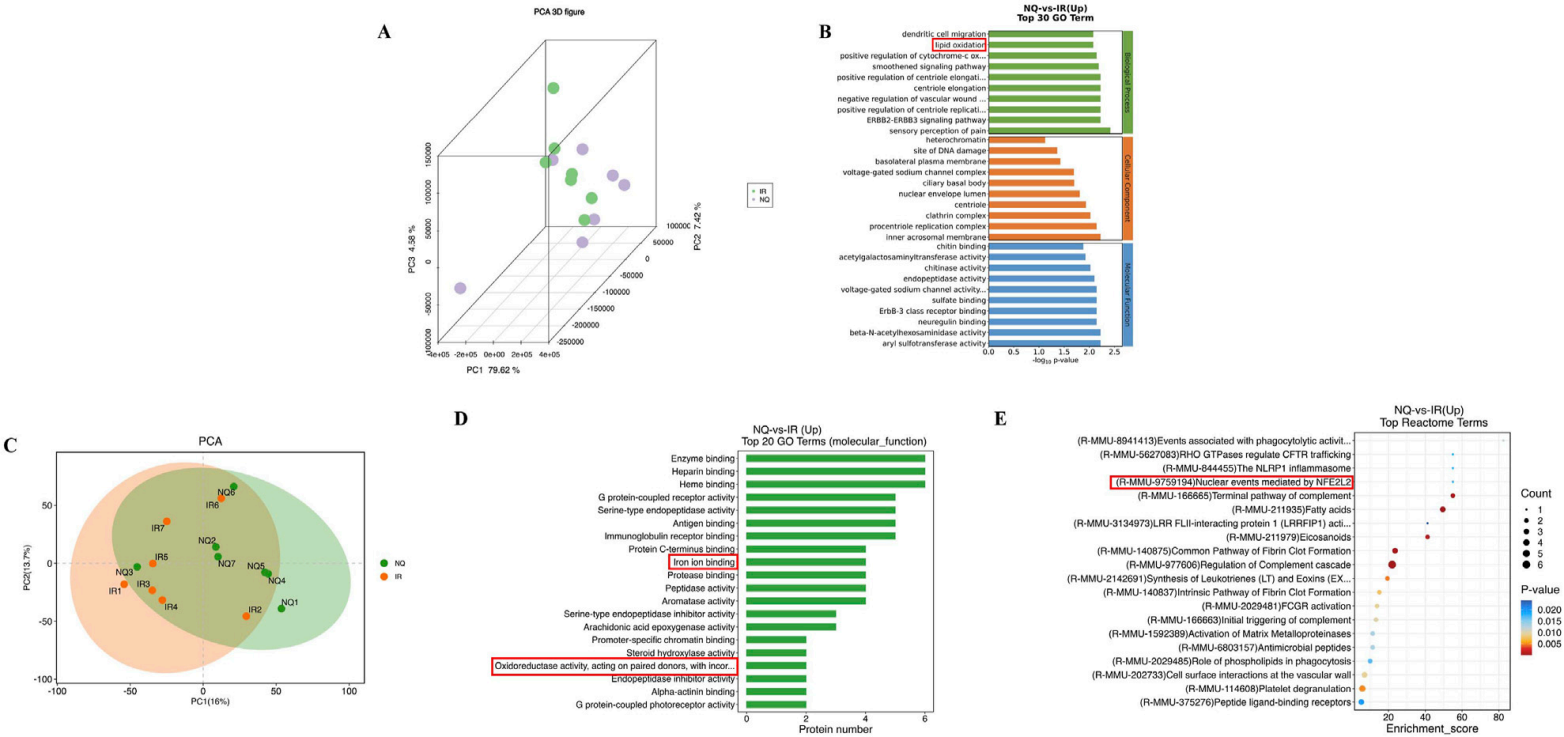
Based on transcriptomic and proteomic analysis, NFE2L2 (Nrf2) is a potential target for NQ to alleviate AIS. **(A)** Principal component analysis (PCA) of the expression of credible genes. Each point in the figure represents a sample, green represents the IR group, and purple represents the NQ group. **(B)** GO enrichment analysis top 30 of NQ group and IR group. **(C)** Principal component analysis (PCA) was performed on the expression of credible proteins. Each point in the figure represents a sample, orange represents the IR group, and green represents the NQ group. **(D)** GO enrichment analysis of molecular function top20 histogram. **(E)** Reactome enrichment analysis top20 bubble plot (n = 7/group).

Our study found that the dose of 520 mg/kg NQ was optimal for the treatment of AIS in mice. To further investigate the protective mechanism of NQ against IS, we conducted a transcriptomic and proteomic analysis and analyzed the differentially expressed genes (DEGs) and differentially expressed proteins (DEPs) in the IR group and 520 mg/kg NQ group (7 samples in each group). DEGs and DEPs were considered as genes with Foldchange ≥ 2 or Foldchange ≤ 1/2 and p-value < 0.05. The expression patterns of DEGs in the IR group and NQ group are shown by principal component analysis (PCA), revealing differences between them (Figure 2A). NQ-vs-IR upregulated DEGs were enriched in GO, and “Lipid oxidation” was significant at the biological process level (Figure 2B). PCA was used to show the expression patterns of all DEPs, which showed significant differences between the IR group and the NQ group (Figure 2C). The GO enrichment analyzed top 20 histogram of molecular function showed that the DEPs were mainly enriched in “Iron ion binding” and “Oxidoreductase activity” (Figure 2D). Additionally, we also enriched the upregulated genes of NQ-VS-IR in Reactome, among which “(R-MMU-9759194) Nuclear events mediated by NFE2L2” was focused (Figure 2E). *Nrf2* plays a crucial role in regulating both iron homeostasis and oxidative stress (Kerins and Ooi, 2018). Combining the results of transcriptomics and proteomics, we believe that NQ ameliorates AIS induced oxidative stress damage and ferroptosis by affecting the *Nrf2* mediated ferroptosis.

### 3.3 NQ inhibits AIS-induced ferroptosis by activating *Nrf2* expression and alleviating lipid peroxidation and iron accumulation

**FIGURE 3:**
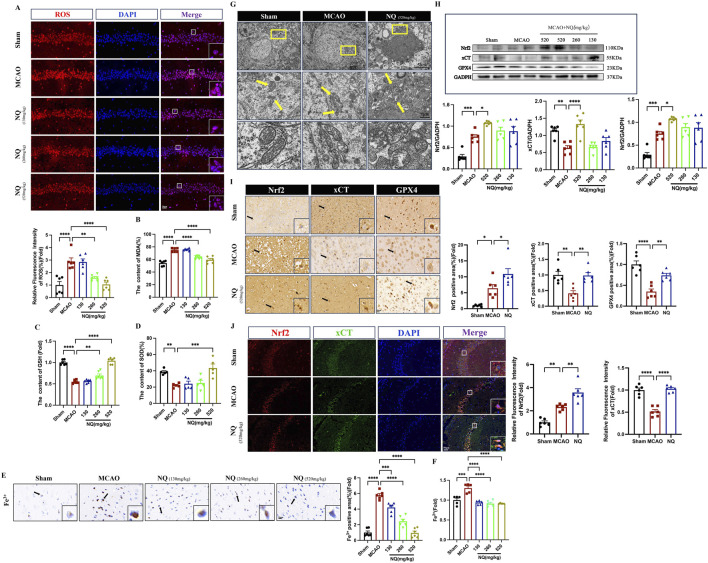
NQ inhibits AIS-induced ferroptosis by activating *Nrf2* expression and alleviating lipid peroxidation and iron accumulation. **(A)** Reactive oxygen species (ROS) immunofluorescence staining was utilized to identify the extent of oxidative stress damage in each subgroup. Microscopy images were obtained to showcase representative samples. (scale bar = 50 μm, n = 6/group). **(B–D)** The malondialdehyde (MDA), glutathione (GSH) ane superoxide (SOD) levels in the brain tissue of mice with AIS (n = 6/group). **(E)** Prussian blue staining to detect iron deposition of Fe^3+^ in the cerebral cortex with AIS mice (scale bar = 20 μm, n = 6/group). **(F)** The Fe^2+^ level in the brain tissue of mice with AIS (n = 6/group). **(G)** Representative transmission electron microscopy (TEM) micrographs depicting mouse hippocampal neurons with CIR. The bottom row features magnified images of the mitochondria (scale bar = 5 μm/1 μm). **(H)** Western blot was performed to investigate the relative density ratios of *Nrf2*, xCT and GPX4 expression in the brain tissue of each group (n = 6/group), using GAPDH as a loading control. The densities of GAPDH, *Nrf2*, xCT and GPX4 proteins were measured with ImageJ software. **(I)** Immunohistochemical staining of SYN (sepia) in the hippocampus (n = 6/group; scale bar = 20 μm). **(J)** Immunofluorescence of the hippocampus co-stained with *Nrf2* (red) and xCT (green) (n = 6/group; scale bar = 50 μm). Bars represent mean ± SEM, statistical analysis was performed using a one-way ANOVA with Tukey’s *post hoc* test. *p < 0.05, **p < 0.01, ***p < 0.001 and ****p < 0.0001.

Lipid peroxidation and iron accumulation are the key events in ferroptosis. To investigate the effect of NQ on ferroptosis, a series of ferroptotic events were examined. In AIS mice, an increase of ferroptotic events was found, including important marker of cellular oxidative damage ROS promotion (Figure 3A), product of lipid peroxidation MDA addition (Figure 3B), important antioxidant enzymes GSH and SOD depletion (Figures 3C, D), as well as Fe^3+^ and Fe^2+^ accumulation (Figures 3E, F). However, ROS, MDA, Fe^3+^, and Fe^2+^ were downregulated and the level of GSH and SOD were increased in ischemic brain tissue after being treated with NQ. Furthermore, the results of transmission electron microscope (TEM) revealed that the mitochondria in the MCAO group exhibited reduced size, increased membrane density and shorter, smaller cristae that moved towards the edge compared to the sham group, indicating that ferroptosis was significantly occurred in the neuronal cells of MCAO-induced AIS. Conversely, there was an improvement in mitochondrial volume crumpling in the NQ treated group (Figure 3G). These results confirmed that NQ inhibits AIS-induced ferroptosis. To further investigate the effect of NQ on ferroptosis, the *Nrf2*, xCT, and GPX4 were measured. Western blotting and immunohistochemistry staining showed that a significant decrease in xCT and GPX4 levels and an increase in *Nrf2* levels was observed in AIS mice, whereas xCT, GPX4 as well as *Nrf2* levels were significantly increased, particularly in the 520 mg/kg NQ group mice (Figures 3H, I). It is commonly acknowledged that *Nrf2* functions as a pivotal transcription factor in the response to oxidative stress. In homeostatic conditions, *Nrf2* is sequestered in the cytoplasm by its binding partner, KEAP1, where it undergoes ubiquitin-dependent degradation mechanisms, thus ensuring the maintenance of its low concentrations. Upon stress, there is a stress-induced increase and translocation of *Nrf2* to the nucleus where it exerts antioxidant effects during the acute phase, which explains why there is an increase in *Nrf2* in AIS mice. More interestingly, immunofluorescent colocalization indicated that *Nrf2* was primarily expressed in the cytoplasm in the sham group, while mainly expressed in the nucleus and colocalizes with xCT in the MCAO and NQ groups (Figure 3J). Furthermore, the expression of *Nrf2* and xCT were consistent with the results of immunohistochemistry and Western blotting. These results indicates that NQ potentially stimulates the activation and nuclear translocation of *Nrf2*, enhancing the expression of xCT and GPX4. This augmentation in expression leads to the exertion of antioxidant stress effects, thus affording protection against ferroptosis in AIS mice.

### 3.4 NQ alleviates Erastin-induced ferroptosis by activating *Nrf2*/xCT/GPX4 pathway *in vitro*

**FIGURE 4:**
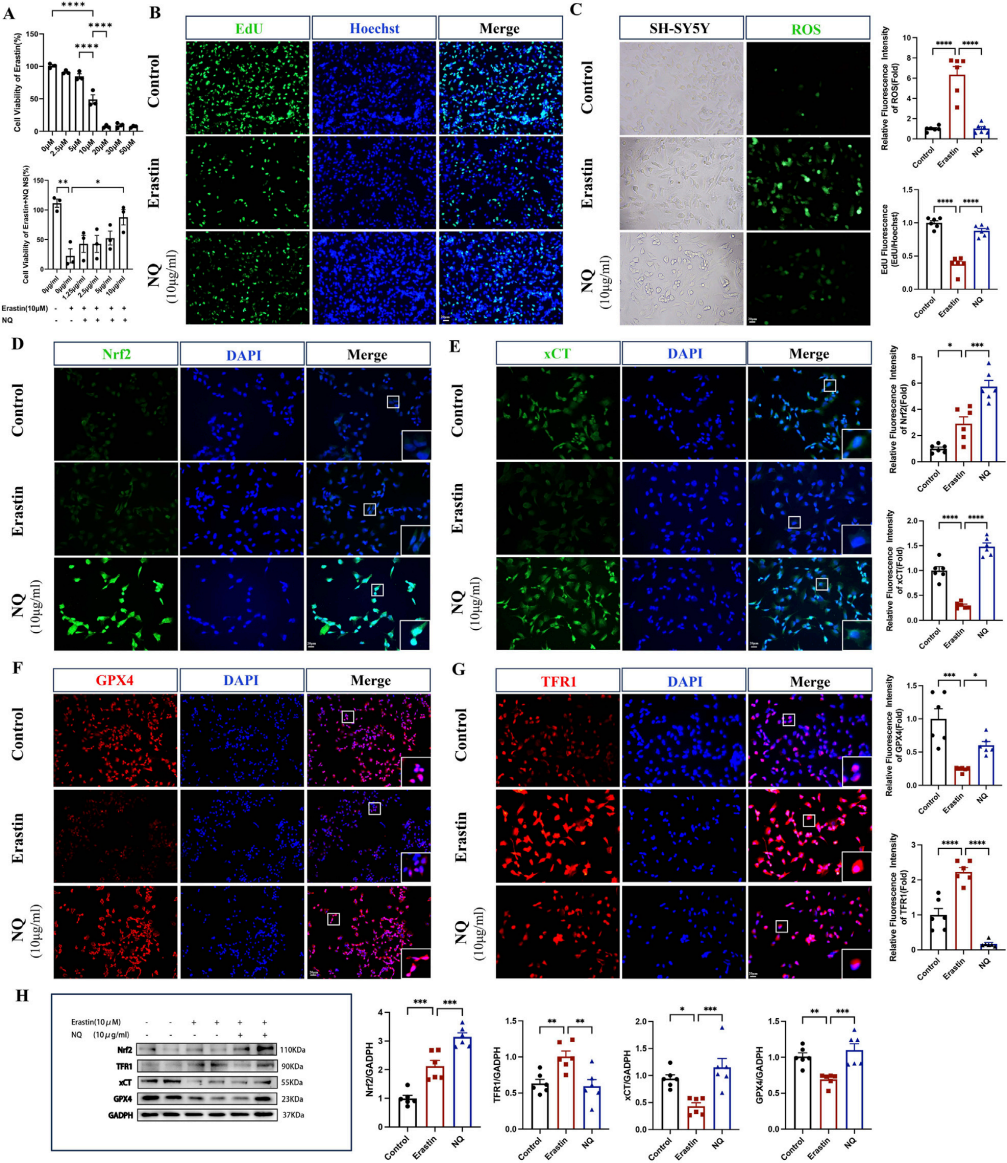
NQ alleviates Erastin-induced ferroptosis by activating Nrf2/xCT/GPX4 pathway *in vitro*. **(A)** Cell viability after administration of Erastin and Erastin + NQ by cck8 assay (n = 3/group). **(B)** Cell proliferation was analyzed using Edu fluorescence, utilizing EdU (green) and Hoechst (blue) staining (scale bar = 20 μm, n = 6/group). **(C)** Lipid peroxidation levels were measured using ROS Fluorescence with ROS (green) (scale bar = 20 μm, n = 6/group). **(D–G)** *Nrf2*, xCT, TFR1 and GPX4 immunofluorescence staining and representative images were captured by fluorescence microscopy (scale bar = 20 μm, n = 6/group). **(H)** Western blot analysis of the relative density ratios of *Nrf2*, xCT, TFR1 and GPX4 expression in the SH-SY5Y of each group (n = 6/group). GAPDH was used as a loading control. Densities of GAPDH, *Nrf2*, xCT, TFR1 and GPX4 proteins were measured using ImageJ software. Bars represent mean ± SEM, statistical analysis was performed by one-way ANOVA with the Tukey’s *post hoc* test. *p < 0.05, **p < 0.01, ***p < 0.001, and ****p < 0.0001.

**FIGURE 5:**
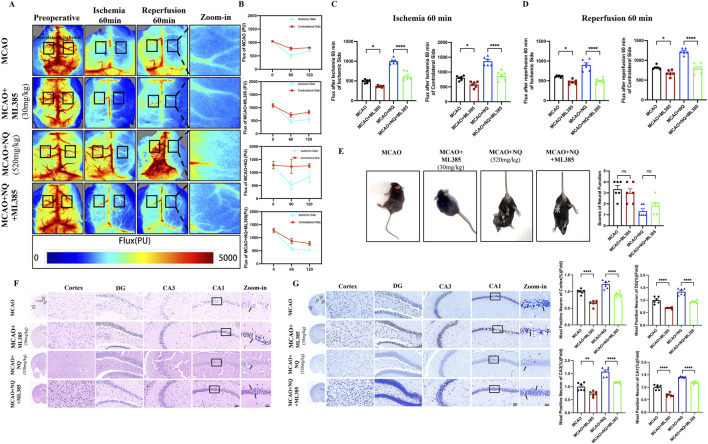
Inhibition of the *Nrf2* pathway weakens the effect of NQ to protect the brain from AIS. **(A)** Representative raw data from LSCI with defined ROIs on the ischaemia and contralateral sides. **(B)** Flux values of ROIs in AIS mice preoperatively, 60 min of ischaemia and 60 min of reperfusion (n = 6/group). **(C)** Differences in flux changes between the ischaemic and contralateral sides at 60 min of ischaemia in the MCAO, ML385, NQ and NQ + ML385 groups (n = 6/group). **(D)** Differences in flux changes between the ischaemic and contralateral sides at 60 min of reperfusion in the MCAO, ML385, NQ and NQ + ML385 groups (n = 6/group). **(E)** Neurobehavioral scores of mice were recorded at the time of postoperative awakening according to the Zea-Longa neurobehavioral score (n = 6/group). **(F)** HE stained sections of the cerebral cortex, hippocampal DG, CA1 and CA3 of representative mice from each group (scale bar = 50 μm/20 μm). **(G)** Nisssl stained sections of the cerebral cortex, hippocampal DG, CA1 and CA3 of representative mice from each group (scale bar = 50 μm/20 μm, n = 6/group). Bars represent mean ± SEM, statistical analysis was performed by one-way ANOVA with the Tukey’s *post hoc* test. *p < 0.05, **p < 0.01, ***p < 0.001, and ****p < 0.0001.

To further elucidate the role of NQ in ferroptosis, the ferroptosis inducer Erastin was used to incubate SH-SY5Y cells to specifically establish cell ferroptosis model. First, SH-SY5Y cells were exposed to different concentrations of NQ, ranging from 0 to 10 μg/mL, for 24 h. The obtained results showed that within this concentration range, no IC50 value was observed and surface NQ was not toxic to cells (Supplementary Material S3). We determined the optimal concentration of Erastin and NQ-treated cells using the cck8 assay. SH-SY5Y cells were treated with varying concentrations of Erastin ranging from 0 to 50 µM for 24 h. The concentration that inhibited 50% of cell viability, IC50, was achieved at 10 µM. The cells were treated with 10 µM Erastin for 24 h, followed by the addition of NQ. The cells exhibited an IC50 at an NQ concentration of 10 μg/mL, which corresponds to the maximum concentration at which NQ lyophilized powder can dissolve in DMSO (Figure 4A). The EdU fluorescence assay demonstrated decreased cell proliferation in the Erastin group compared to the control. In the NQ group, EdU (green) fluorescence intensity exceeded that of the Erastin group, indicating an increase in cell proliferation (Figure 4B). After Erastin (10 μM) treatment, significant ferroptosis was induced in SH-SY5Y cells with ROS increasing. Attractively, the production of ROS could be abolished in ferroptotic cell by 10 μg/mL NQ (Figure 4C). These findings suggest that NQ is effective in reducing ferroptosis. Additionally, quantifications of *Nrf2*, xCT, GPX4 and TFR1 protein expression was performed by immunofluorescence and Western blotting after Erastin incubation. The NQ group displayed a significant increase in *Nrf2* in the nucleus, indicating the activation of *Nrf2*’s antioxidant capacity (Figures 4D, H). Moreover, Erastin blocked the xCT and GPX4 expression, and elevated TFR1 expression, implying the alteration of crucial protein levels in ferroptosis. Attractively, NQ completely revised xCT, GPX4 and TFR1 expression in the induction of ferroptosis (Figures 5E–H). Thus, based on the findings above, we suggest that NQ inhibits ferroptosis induced by Erastin by activating *Nrf2*/xCT/GPX4 pathway.

### 3.5 Inhibition of the *Nrf2* pathway weakens the effect of NQ to protect the brain from AIS

To investigate the role of *Nrf2* in protection against AIS of NQ, *Nrf2* inhibitor ML385 was used to inhibit the activation of *Nrf2*. The CBF decreased at 60 min of ischemia and increased but not to normal levels at 60 min of reperfusion in MCAO, MCAO + ML385, NQ and NQ + ML385 groups (Figures 5A, B). After 60 min of ischemia, both ipsilateral and contralateral CBF in the MCAO + ML385 group were lower than those in the MCAO group (Figure 5C). Following 60 min of reperfusion, the ipsilateral CBF in the MCAO + ML385 group remained lower than that in the MCAO group, indicating that ML385 can reduce cerebral blood flow in AIS mice (Figure 5D). Notably, at both 60 min of ischemia and 60 min of reperfusion, the CBF in the NQ + ML385 group was lower than that in the NQ group, suggesting that inhibition of *Nrf2* expression can counteract the NQ-induced increase in CBF (Figures 5C, D). AIS mice exhibited signs of consciousness loss, contralateral paralysis, and inability to walk. However, post-administration of ML385, there was no significant change in the extent of limb damage in both MCAO and NQ mice, which may be because the severity of limb damage is related to whether the brain regions governing motor functions are affected, and less related to other interventional factors (Figure 5E). HE staining revealed that neuronal cytolysis and crumpled nuclei in in the cerebral cortex and hippocampal regions both in MCAO and MCAO + ML385 groups. Compared with the NQ group, the NQ + ML385 group displayed more crumpled nuclei (Figure 5F). Nissl staining revealed a reduction in Nissl vesicles in AIS mice following ML385 intervention in comparison to the MCAO group. Similarly, a reduction in Nissl vesicles was observed in the NQ + ML385 group compared with the NQ group (Figure 5G). The results suggest that inhibition of *Nrf2* expression may exacerbate neuronal damage in AIS mice, and the neuroprotective effect of NQ appears to be mediated through the *Nrf2* pathway.

### 3.6 Inhibition of *Nrf2* leads to reduced resistance to ferroptosis in NQ

**FIGURE 6:**
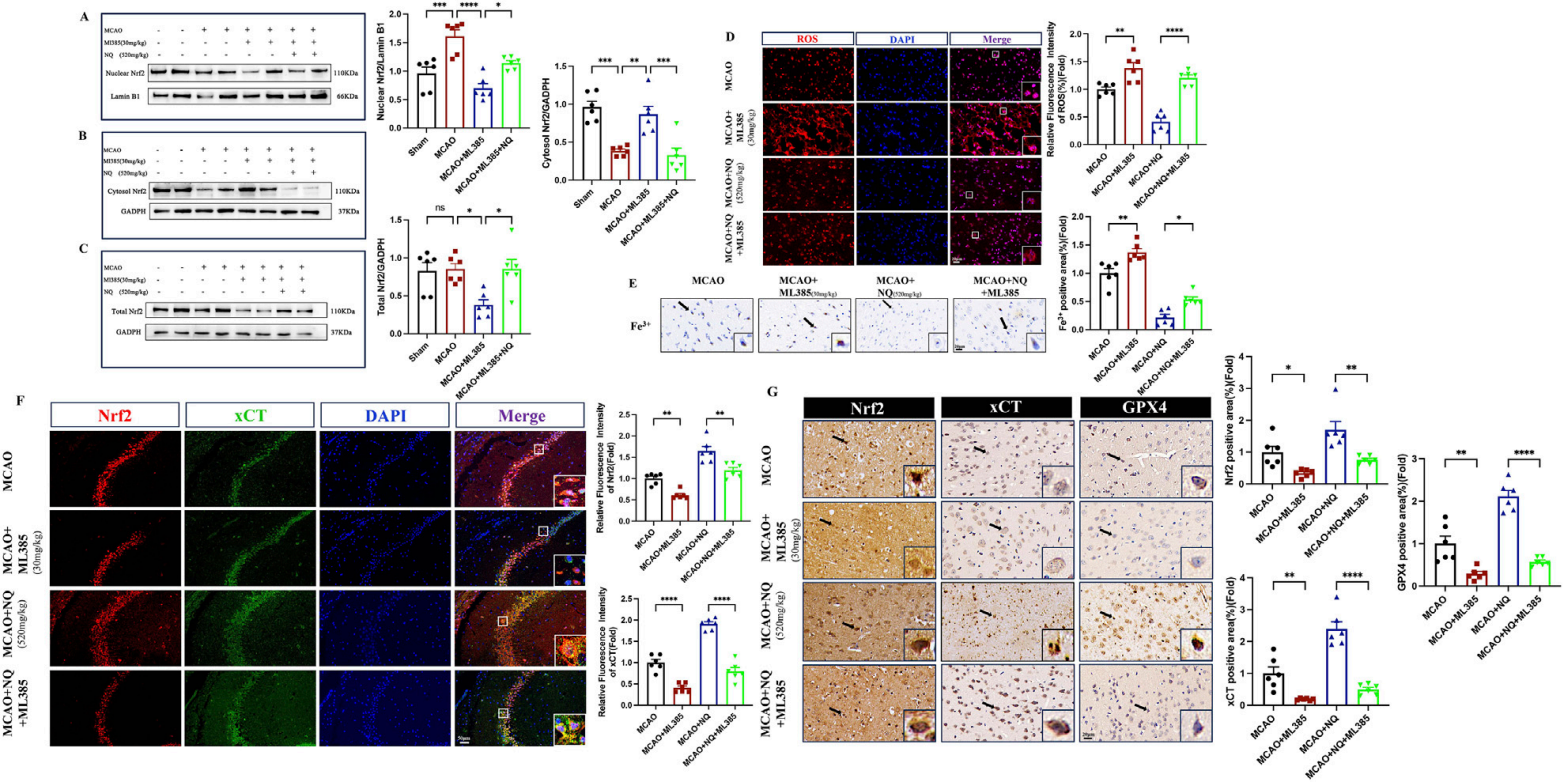
Inhibition of *Nrf2* leads to reduced resistance to ferroptosis of NQ. **(A**–**C)** Western blot analysis of the relative density ratios of nuclear *Nrf2*, cytosol *Nrf2* and total *Nrf2*, and their corresponding expressions in the brain’s tissue of each group (n = 6/group). GAPDH was used as a loading control for total *Nrf2* and cytosol *Nrf2*. Lambin B1 was used as a loading control for nuclear *Nrf2*. Densities of GADPH, Lamin B1, total *Nrf2*, cytosol *Nrf2* and nuclear *Nrf2* proteins were measured using ImageJ software. **(D)** ROS immunofluorescence staining was conducted to identify the level of oxidative stress damage in each subgroup. Subsequently, representative images were obtained using fluorescence microscopy (scale bar = 20 μm, n = 6/group). **(E)** Prussian blue staining to detect iron deposition of trivalent iron ions in the cerebral cortex (scale bar = 20 μm, n = 6/group). **(F)** Immunofluorescence of hippocampus co-stained with *Nrf2* (red) and xCT (green) (scale bar = 50 μm, n = 6/group). **(G)** Immunohistochemical staining of SYN (sepia) in the cerebral cortex (scale bar = 20 μm, n = 6/group). Bars represent mean ± SEM, statistical analysis was performed by one-way ANOVA with the Tukey’s *post hoc* test. *p < 0.05, **p < 0.01, ***p < 0.001, and ****p < 0.0001.

To investigate the effect of NQ on ferroptosis after inhibiting *Nrf2*, a series of ferroptosis events were examined. Western blot showed that the total protein and nuclear protein levels of *Nrf2* in AIS mice were significantly increased, while the cytoplasmic protein levels were low. The total *Nrf2* protein and nuclear protein levels in the MCAO + ML385 group were significantly lower than those in the MCAO group, and the cytoplasmic protein levels were increased, indicating that activation of *Nrf2* was inhibited by ML385 in AIS mice. Compared with the ML385 group, the ML385 + NQ group showed significantly higher levels of total *Nrf2* protein and nuclear protein, and lower levels of cytoplasmic protein, suggesting that NQ was still partially able to activate *Nrf2* after treatment with ML385 (Figures 6A–C). This also elucidates why NQ continues to exert a certain protective effect on AIS even after inhibition with *Nrf2*. ROS and Fe^3+^ in the ML385 group were higher than the MCAO group, while the NQ + ML385 group exhibited an accumulation of ROS and Fe^3+^ compared to the NQ group (Figures 6D, E). Immunohistochemical analysis and immunofluorescence techniques revealed a marked downregulation in the expressions of *Nrf2*, xCT, and GPX4 in AIS mice following ML385 intervention. Similarly, a significant reduction in the expression levels of *Nrf2*, xCT, and GPX4 was observed in the NQ + ML385 group in comparison to the NQ group alone (Figures 6F, G). These findings indicate that ML385 inhibited the activation of *Nrf2*, downregulated the expression of xCT and GPX4, and exacerbated oxidative stress and iron deposition induced by MCAO, and weakened NQ’s resistance to ferroptosis. Immunofluorescence co-localisation showed that *Nrf2* was expressed in both the nucleus and cytoplasm in the MCAO group, while *Nrf2* was mainly expressed in the cytoplasm in the ML385 group. *Nrf2* was expressed in both the nucleus and the cytoplasm in the NQ group, while *Nrf2* was mainly expressed in the cytoplasm in the NQ + ML385 group. These results indicated that ML385 inhibited *Nrf2* activation in AIS mice (Figures 6F, G). In summary, the effect of NQ in attenuating AIS-induced ferroptosis is diminished after inhibition of *Nrf2*, suggesting that NQ inhibits the occurrence of ferroptosis in AIS mice via activating the *Nrf2* pathway.

## 4 Discussion

Through a combination of *in vitro* and *in vivo* analyses, we discovere that NQ influences the ferroptosis pathway involving xCT/GPX4 by activating *Nrf2*, thereby mitigating neuronal ferroptosis triggered by AIS. Our study represents the inaugural investigation into the impact of *Nrf2* on neuronal ferroptosis induced by AIS and sheds light on the molecular mechanisms underlying NQ’s therapeutic effect in the treatment of IS.

Thrombolytic therapy, the primary clinical approach to treating AIS, aims to restore blood recanalisation but may potentially worsen brain injury (Molina, 2011). Understanding the AIS mechanism and developing medications to prevent CIRI after post-thrombolysis blood recanalisation remains crucial. Our findings indicate that the administration of NQ effectively preserved neural function, significantly reducing the size of brain infarcts and pathological lesions observed *in vivo*. AIS is a multifaceted pathological process involving various mechanisms. Recent research implicates ferroptosis as a crucial factor in AIS post-stroke (Tuo et al., 2022; Yu et al., 2021). Through transcriptomic and proteomic bio-enrichment analysis, we find that AIS causes abnormalities in lipid and iron metabolism and disrupts iron homeostasis. TEM revealed mitochondrial alteration associated with ferroptosis post-I/R injury, mitigated significantly after NQ administration (morphologically spherical mitochondria, intensified membrane thickness and reduced crest). Iron is found mainly in the form of Fe^3+^ bound to transferrin. It enters cells by endocytosis, which is facilitated by the binding of transferrin receptor 1 (TFR1) to the cell membrane. In the body, Fe^3+^ undergoes a redox reaction to form Fe^2+^. Unstable Fe^2+^ catalyses the Fenton reaction, forming ROS and inducing lipid peroxidation and ultimately leading to iron toxicity (Koppula et al., 2021). The administration of NQ in this study led to a significant decrease in ROS levels, MDA levels, which is a by-product of lipid peroxidation, Fe^2+^ and Fe^3+^which is involved in the Fenton reaction. Additionally, significant increasing in the antioxidant enzymes were SOD and GSH. The association between NQ and ferroptosis was further validated by investigating the expression of proteins related to this process, such as xCT and GPX4. NQ significantly upregulated xCT, GPX4 and *Nrf2* protein. In ferroptosis cell model SH-SY5Y cells, after NQ intervention, ROS levels were significantly reduced, xCT, GPX4 and *Nrf2* levels were increased, and TFR1 levels were decreased. These findings suggest that the potential neuroprotective effect of NQ against AIS could be attributed to its ability to inhibit iron metamorphosis. Nevertheless, the correlation between this increased *Nrf2* level and the inhibitory effect of NQ on ferroptosis necessitates further investigation.

Nuclear factor E2-related factor 2 (*Nrf2*), a transcription factor, plays a pivotal role in regulating diverse cellular functions such as endogenous antioxidant defense, ferroptosis, and lipid metabolism processes (Torrente and DeNicola, 2022). *Nrf2* also plays a neuroprotective role in multiple diseases such as stroke, Alzheimer’s disease and Parkinson’s disease (Delaidelli et al., 2021). Several ferroptosis-related genes, namely GSH, xCT and GPX4, are regulated by *Nrf2* transcription (Fu et al., 2022). Studies have shown that the *Nrf2* agonist Oltipraz can alleviate and protect IS by inhibiting oxidative stress and ferroptosis (Jian et al., 2024). Our study revealed that NQ can enhance *Nrf2* activity. We administered the *Nrf2* inhibitor ML385 to validate the protective mechanism of NQ against AIS. ML385 is a specific inhibitor of the *Nrf2* protein and functions in the *Nrf2*/ARE pathway which is involved in the regulation of antioxidant, as well as in maintaining the integrity of the BBB (Cui et al., 2021). The findings suggest that ML385 attenuates the protective capabilities of NQ against AIS, resulting in elevated ROS levels and downregulation of *Nrf2* as well as ferroptosis-associated proteins, namely xCT and GPX4. These findings suggest that NQ has a protective impact on AIS, which could be accomplished by alleviating ferroptosis through the *Nrf2* pathway. Consistent with previous studies, activation of the PI3K/AKT/Nrf2 and xCT/GPX4 signaling pathways can improve cerebral ischemic injury (Fu et al., 2022), and activation of the Nrf2/xCT/GPX4 signaling pathway can prevent OGD/R-induced iron overload (Yuan et al., 2021). Studies have shown that ferroptosis in *Nrf2* ^−/−^ mice is significantly aggravated and GPX4 expression is downregulated (Huang S. et al., 2022), so whether *Nrf2* alleviates IS-induced ferroptosis by regulating the xCT/GPX4 pathway aroused our interest.

However, this study also has some limitations. In future studies, we will further explore the mechanism of action of NQ based on the active ingredient moieties of other herbs in the compound formulas. In addition, we will further investigate the mechanism of NQ regulation of xCT/GPX4 pathway through *Nrf2* on mice cortical neurons and *Nrf2* knockout mice. Studies have shown that activation of *Nrf2* can inhibit ACSL4-mediated lipid peroxidation and ferroptosis (Fu et al., 2024), whether NQ protects IS through this pathway is also worthy of further study. Ferroptosis also regulates through the FSP1-COQ0-NADPH pathway, E-cadherin-*Nrf2*-Hippo-YAP signaling, AMPK signaling (Jiang et al., 2021), and Whether NQ is involved in regulating other ferroptosis pathways and whether NQ is involved in regulating key ferroptosis proteins such as ACSL4, FTH1, and FTL are also topics for our future research.

## 5 Conclusion

Our results show that NQ can activate the xCT/GPX4 pathway mediated by *Nrf2* to alleviate AIS -induced neuronal cell ferroptosis. This study provides the first scientific evidence to support NQ as a potential preventive medicine for AIS.

## Data Availability

The datasets presented in this article are not readily available because of confidentiality agreements. Requests to access the datasets should be directed to jianting9388@126.com.

## Glossary

AIS: acute ischaemic stroke
NQ: Naoqing formula
CIRI: cerebral ischaemia/reperfusion injury
GPX4: glutathione peroxidase 4
SLC7A11: cystine/glutamate transporter system xC-/xCT
ROS: reactive oxygen species
*Nrf2*: Nuclear factor-erythroid 2 (NF-E2)-related factor 2
DG: dentate gyrus
CA: cornu amoni
MCAO: Middle Cerebral Artery Occlusion
TTC: triphenyl tetrazolium chloride
GC: gas chromatography
ROI: region of interest
CCA: common carotid artery
ECA: external carotid artery
ICA: internal carotid artery
CBF: cerebral cortex blood flux
LSCI: laser speckle contrast imaging
PCA: principal component analysis
DEG: differently expression gene
DEP: differently expression protein
TEM: transmission electron microscopy
MDA: malondialdehyde
GSH: glutathione
SOD: superoxide dismutase
IHC: Immunohistochemistry
IF: Immuno-fluorescence
PVDF: polyvinylidene fluoride
TFR: transferrin receptor
MS: Mass spectrometry
IS: ischemic stroke
CNS: central nervous system
TCM: raditional Chinese medicine
