# Supplementary material for: Naoqing formula alleviates acute ischaemic stroke-induced ferroptosis via activating Nrf2/xCT/GPX4 pathway
Source: Front Pharmacol. 2024 Dec 17;15:1525456. doi: 10.3389/fphar.2024.1525456 (PMC11686226; doi:10.3389/fphar.2024.1525456)
Supplement: Supplementary file 1 [file DataSheet3.docx]

Supplementary Material 3


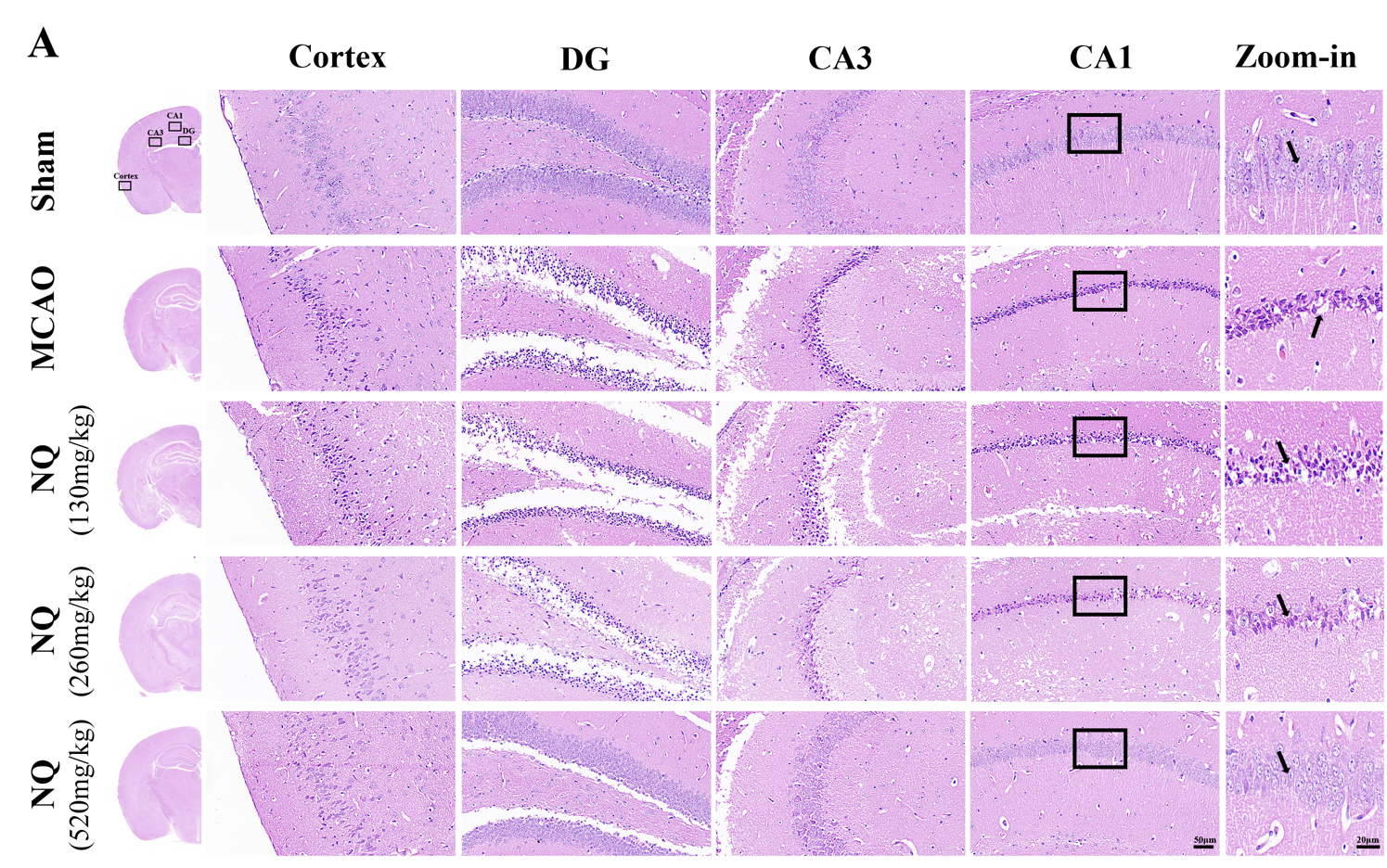


HE stained sections of the cerebral cortex, hippocampal DG, CA1 and CA3 of representative mice from each group (scale bar=50μm/20μm).

To assess the cellular toxicity of NQ, we conducted a CCK-8 assay. For this purpose, SH-SY5Y cells were exposed to various concentrations of NQ, spanning from 0 to 10µg/ml, for a duration of 24 hours. The obtained results indicated that within this concentration range, no IC50 value was observed. 10 µg/ml is the maximum concentration of NQ lyophilised powder dissolved in DMSO.
