# Supplementary material for: Naoqing formula alleviates acute ischaemic stroke-induced ferroptosis via activating Nrf2/xCT/GPX4 pathway
Source: Front Pharmacol. 2024 Dec 17;15:1525456. doi: 10.3389/fphar.2024.1525456 (PMC11686226; doi:10.3389/fphar.2024.1525456)
Supplement: Supplementary file 2 [file DataSheet2.docx]

Supplementary Material 2

**Materials and method**

**1.1 Neurologic deficit scoring**

After 24 hours of reperfusion, neurological deficit scores were assessed by experienced investigators using the Longa method^17.^ A double-blind approach was adopted to minimise bias. Neurological deficit scores were recorded as follows: 0 indicated no neurological deficits; 1 indicated difficulty straightening the contralateral forelimb; 2 indicated turning to the opposite side while crawling; 3 indicated walking the body to the opposite side; and 4 indicated an inability to walk independently, leading to loss of consciousness.

**1.2 TTC staining**

The mice were euthanised using 2% pentobarbital sodium, followed by decapitation. The brains were subsequently removed to measure infarct volume. Coronal brain sections were stained with 2% TTC (Sigma, BCBP3272V) at 37°C for 20 min. Digital images of the slices were captured and analysed using ImageJ software (National Institutes of Health, USA) to calculate cerebral infarct volume.

**1.3 Hematoxylin-eosin (H&E) staining**

Brain tissues were fixed in 4% paraformaldehyde for a day. Following this, the brain tissue was dehydrated in varying concentrations of ethanol and xylene and embedded in paraffin. Paraffin sections were stained with H&E and examined under a light microscope for cerebral cortex and hippocampal histology analysis.

**1.4 Nissl staining**

Tissue sections were treated with a dye solution for 2-5 min, followed by rinsing with tap water. A 0.1% solution of glacial acetic acid was employed (the reaction ceased on washing with running water) for slight differentiation, controlled under a microscope. The sections were then washed with tap water and oven-dried. The hippocampus was observed under a microscope to determine any morphological changes, and neuronal counting was used to record the number of surviving neurons.

**1.5 4D-DIA quantitative proteomic analysis**

The mice were euthanized using 2% sodium pentobarbital, and the brain tissue was carefully removed and stored in a refrigerator at -80◦C. Finally, the samples stored in dry ice were transported to Shanghai OE biotech company for protein mass spectrometry analysis. Proteins with Foldchange≥2or Foldchange≤1/2 & p-value<0.05.

**1.6 Transcriptome sequencing**

Total RNA was extracted from brain tissue using a mirVana miRNA Isolation Kit (Ambion, Austin, TX, USA). RNA integrity was assessed using an Agilent 2100 Bioanalyzer (Agilent Technologies, Santa Clara, CA, USA). Libraries were constructed using the TruSeq Stranded mRNA LT Sample Prep Kit (Illumina, San Diego, CA, USA) according to the manufacturer's instructions. An Agilent 2100 Bioanalyzer was used to determine library size and purity. These libraries were sequenced on an Illumina sequencing platform (HiSeqTM 2500 or Illumina HiSeq X Ten) and 125/150 bp paired-end reads were generated. Clean reads were mapped to the mouse reference genome (GRCm38) using HISAT2. FPKM values for each gene were calculated using Cufflinks, and read counts for each gene were obtained using htseq-count. RNA-seq was performed by OE Biotech Co., Ltd. (Shanghai, China).

**1.7 Transmission electron microscopy (TEM)**

Damaged hippocampi ultrastructure was analysed using transmission electron microscopy (TEM, Hitachi, HT7800/HT7700). The ischaemic cortical tissue was sliced into 1 mm3 cubes, fixed with glutaraldehyde (2.5%) and osmic acid (1%), and processed for dehydration, embedding, slicing and staining with lead citrate and uranyl acetate. The copper grids were examined under TEM to capture images.

**1.8 Isolation and preparation of nuclear and cytoplasmic proteins**

All procedures adhere to the guidelines provided by the Nuclear and Cytoplasmic Protein Extraction Kit (GBCBIO, China).

**1.9Oxidative stress indices SOD MDA and GSH level**

Ischaemic tissue homogenates were used to determine malondialdehyde (MDA) production and reduced glutathione (GSH) levels following the instructions provided for MDA (Beyotime, China, S0131S), GSH (Solarbio, China, BC1175) and superoxide dismutase (SOD) (Solarbio, China, BC0175).

**1.10 Iron measurements**

Fe^2+^ concentrations were assessed using the iron assay kit (Elabscience, China) per the manufacturer’s instructions.

**1.11 Perls staining + DAB staining**

Slides were submerged in a mixture of 2% potassium ferrohydrate and 2% hydrochloric acid, stained for 30 min and washed twice with distilled water. After staining the slides with DAB solution for approximately 5-10 min, the degree of colour development was observed and controlled under a microscope.. Upon examination, the tissue's iron-containing components appeared brown, while the cell nucleus displayed a light blue shade.

**1.12 CCK-8 assay**

Cell proliferation was analysed using the CCK-8 assay (GBCBIO, China) as described by the manufacturer. Each assay was performed in triplicate.

**1.13 Edu assay**

The EdU assay was used to assess cell viability using the BeyoClick™ EdU Cell Proliferation Kit with Alexa Fluor 488 (Beyotime, China). The cells were then incubated with EdU for 2 hours, fixed with 4% paraformaldehyde for 15 minutes and permeabilized with 0.3% Triton X-100 for 15 minutes. The cells were incubated with the click reaction mixture for 30 minutes at room temperature in the dark, followed by incubation with Hoechst for 10 minutes. Fluorescence microscopy was used for detection.

**1.14 Intracellular reactive oxygen species (ROS) detection**

Intracellular reactive oxygen species (ROS) were quantified using the fluorescent probe DCFH-DA. Cells were incubated in 10M DCFH-DA (Beyotime, China) for 30 minutes at 37°C in the dark. The cells were then washed three times with PBS and analysed by fluorescence microscopy.

**1.15 Immunohistochemistry and immunofluorescence**

Paraffin-embedded brain sections were produced from diverse groups designated for this staining process. Immunohistochemistry (IHC) involved dewaxing and rehydrating brain sections through a descending alcohol series coupled with antigen retrieval by sodium citrate buffer. Brain sections were subsequently incubated with specific primary antibodies overnight at 4 ◦C after being blocked with 5% BSA and 3% H2O2 in PBS for 1 hour at room temperature. Subsequently, the sections underwent incubation with HRP conjugates and were subjected to diaminobenzidine detection. The Immunofluorescence (IF) staining procedure followed the same pattern as IHC, with the exception of the processes subsequent to incubation with the appropriate Alexa Fluor 488-labeled or Alexa Fluor 594-labeled secondary antibodies (Bioworld, China). After incubation with DAPI solution, the samples were observed through a fluorescence microscope.

**1.16 Western blot**

Cells and brain tissues were lysed using RIPA buffer (GBCBIO, China) that contained 1 mM PMSF and phosphate inhibitor cocktails, and the collected total proteins were subjected to SDS-PAGE separation (2μlg). The separated proteins were subsequently transferred onto a polyvinylidene fluoride (PVDF) membrane (Millipore, Ireland) and blocked using 5% BSA. The corresponding primary antibodies were then incubated with the membrane overnight at 4℃. After washing with TBST, the membrane was incubated with anti-mouse or anti-rabbit secondary antibodies (Proteintech, China) conjugated with horseradish peroxidase at room temperature for 1 hour. Protein bands were detected using an enhanced chemiluminescence (ECL) substrate kit and analyzed using Image J software. The primary antibodies used in this study were obtained from Proteintech (China). *Nrf2* (16396-1-AP, 1:1000), SLC7A11/xCT (26864-1-AP, 1:500), GPX4 (67763-1-lg, 1:1000), CD71/TFR1 (66180-1-lg, 1:1000), GADPH (6004-1-lg, 1:5000) and LaminB1 (12987-1-AP, 1:5000).
