# Supplementary material for: Naoqing formula alleviates acute ischaemic stroke-induced ferroptosis via activating Nrf2/xCT/GPX4 pathway
Source: Front Pharmacol. 2024 Dec 17;15:1525456. doi: 10.3389/fphar.2024.1525456 (PMC11686226; doi:10.3389/fphar.2024.1525456)

Supplementary Material 1

## Materials and methods

**1.1 Determination of muscone content in Naoqing formula by GC**

An appropriate amount of muscone reference substance was measured and placed in a 50ml brown volumetric flask. Absolute ethanol was added to dilute it and prepare a 0.24 mg/ml muscone reference substance stock solution. Then, 1.0 ml of the microemulsion sample was measured and mixed with 1.0 ml of absolute ethanol. The mixture was ultrasounded for 30 minutes, left to stand for 1 hour, and any lost weight was compensated for using absolute ethanol. The test solution was obtained by filtering with a 0.45 μm filter membrane and using the remaining filtrate. Technical term abbreviations were explained when first used. The language used was clear, objective, and value-neutral, with a formal register and precise word choice. The text was free from grammatical errors, spelling mistakes, and punctuation errors. No changes in content were made. Reference substance solutions of varying concentrations were prepared by measuring 0.25, 0.5, 1.0, 1.5, and 2.0ml of the muscone reference substance stock solution into 5ml brown volumetric flasks and diluting with absolute ethanol. The chromatographic column's temperature followed a programmed temperature rise: it started at 140°C for 8 minutes, then increased to 200°C at a rate of 30°C/min for 10 minutes. The inlet temperature was set to 250°C, the detector temperature to 280°C, and nitrogen was used as the carrier gas. The flow rate was 5ml/min, with a split ratio of 20:1 and an injection volume of 1μl. **Under these conditions, the number of theoretical plates calculated based on the muscone peak was greater than 10,000, and the resolution was greater than 1.5. The peak area was measured six times, with 1μl each time, using the 48μg/ml reference solution from the muscone standard curve.**

**2 Results**

**2.1 Composition analysis and quality control of NQ**

To ascertain the active constituents of NQ and perform quality assurance, we carried out UHPLC-QE-MS and GC-MS analyses. In Fig.1, it depicted the positive **(Fig.1a)** and negative **(Fig.1b)** modes of UHPLC-QE-MS chromatograms. It showed the ion chromatogram of GC-MS**(Fig.1c)**. UHPLC-QE-MS results analyzed 932 compounds, and the main compounds are shown in **Table 1**. GC-MS results analyzed 2645 volatile substances, and the main compounds are shown in **Table 2**.

**2.2 Muscone content in NQ**

The negative solution does not have an interference peak at the position of muscone, and neither excipients nor reagents interfered with the determination of muscone **(Fig.2a)**. Both the GC of the muscone reference solution and the GC of NQ indicated the presence of muscone **(Fig.2b-c)**. A standard curve can be drawn on the ordinate by taking the concentration of the reference solution as the abscissa and the average peak area as the value **(Fig. 2d)**. The regression equation can then be calculated. The results indicated that muscone has a good linear relationship in the concentration range of 12-96μg/ml. The precision of injection and reproducibility of results for determining muscone content was good **(Table 3-4)**. The test solution was prepared according to the specified method for three batches of NQ samples, and the content of muscone was determined by injecting each batch three times **(Table 5)**.

**Table 1 Identification of some compounds of NQ with** **UHPLC-QE-MS**

| **Compound Name** | **Class** | **Formula** | **Mzmed** | **Rtmed** |
| --- | --- | --- | --- | --- |
| Androstenedione | Terpenoids | C19H26O2 | 287.200 | 385.578 |
| Thymol |  | C10H14O | 151.111 | 430.654 |
| Camphor | Terpenoids | C10H16O | \| 153.127 \| \| --- \| | 374.353 |
| Ligustilide | Dihydrofurans | C12H14O2 | 191.106 | 462.502 |
| Senkyunolide A | Miscellaneous | C12H16O2 | 193.122 | 402.044 |
| Levistilide A | Terpenoids | C24H28O4 | 381.206 | 621.626 |
| beta-Asarone | Phenylpropanoids | C12H16O3 | 209.117 | 441.396 |
| Methylisoeugenol |  | C11H14O2 | 179.106 | 329.418 |
| Palmitic Acid | Aliphatic acyl | C16H32O2 | 255.233 | 781.678 |
| Ginsenoside Rg5 | Terpenoids | C42H70O12 | 811.486 | 579.7755 |
| Notoginsenoside Fe | Terpenoids | C47H80O17 | 961.539 | 457.232 |
| 3-[(Carboxycarbonyl)amino]-L-alanine | Alkaloids | C5H8N2O5 | \| 177.050 \| \| --- \| | 32.4913 |
| (-)-Menthone | Isopentenol lipids | C10H18O | 155.143 | 472.033 |
| P-Mentha-1,3,8-triene |  | C10H14 | 135.116 | 357.918 |
| Ginsenoside Rg3 | Terpenoids | C42H72O13 | 783.486 | 487.0285 |

**Table2 Identification of some compounds of NQ with** **GC-MS**

| **Compounds Name** | **Unique Mass** | **R.T. (minutes)** |
| --- | --- | --- |
| Muscone | 195 | 34.8314 |
| Cholesterol | \| 194 \| \| --- \| | 39.6599 |
| Endo-Borneol | \| 119 \| \| --- \| | 23.5976 |
| Isoborneol | \| 28 \| \| --- \| | 23.8907 |
| Camphor | 209 | 19.1244 |
| Humulene | \| 80 \| \| --- \| | \| 23.2067 \| \| --- \| |
| Caryophyllene oxide | \| 152 \| \| --- \| | 29.4433 |
| Isolongifolol | \| 191 \| \| --- \| | \| 32.2018 \| \| --- \| |
| Cis-ligustilide | 190 | 42.0808 |
| Asarone | 208 | 37.9675 |
| Methyleugenol | 147 | 30.0252 |
| Calacorene | 157 | 18.2551 |
| (-)-Neomenthylacetate | 141 | 20.5681 |

**Table3 Result of muscone precision**

| **Sample** | 1 | 2 | 3 | 4 | 5 | 6 | RSD (%) |
| --- | --- | --- | --- | --- | --- | --- | --- |
| **Peak area** | 255.665 | 254.896 | 256.286 | 255.389 | 256.921 | 255.958 | 0.002 |

**Table4 Result of muscone reproducibility**

| **Sample** | **Peak area 1** | **Peak area 2** | | **Peak area 3** | **Average**  **peak area** | **Average peak area** | **RSD (%)** |
| --- | --- | --- | --- | --- | --- | --- | --- |
| 1 | 140.712 | | 141.255 | 140.985 | 140.984 | 139.893 | 0.017 |
| 2 | 138.546 | | 139.129 | 138.086 | 138.587 |  |  |
| 3 | 142.815 | | 143.597 | 143.821 | 143.411 |  |  |
| 4 | 137.385 | | 136.952 | 136.853 | 137.063 |  |  |
| 5 | 139.982 | | 138.519 | 139.752 | 139.418 |  |  |

**Table5 Muscone content of NQ**

| **Batches** | **Muscone content (μg/ml)** | **RSD (%)** |
| --- | --- | --- |
| 1 | 55.49 | 0.48 |
| 2 | 54.26 | 0.56 |
| 3 | 53.64 | 0.45 |

**
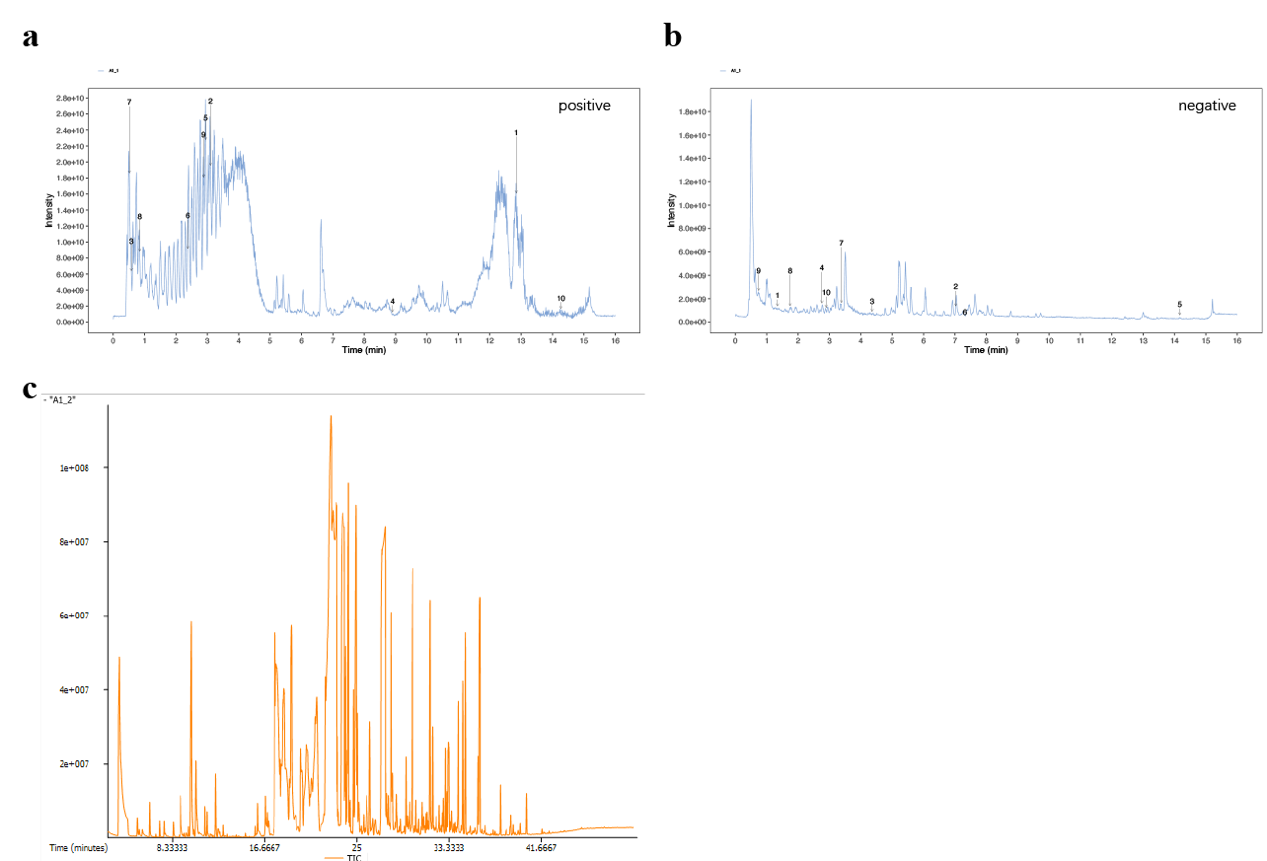
**

**Fig.1. Composition analysis and quality control of NQ**

**(a)**Positive and **(b)** negative mode of UHPLC-QE-MS chromatograms. **(c)** The ion chromatogram of GC-MS


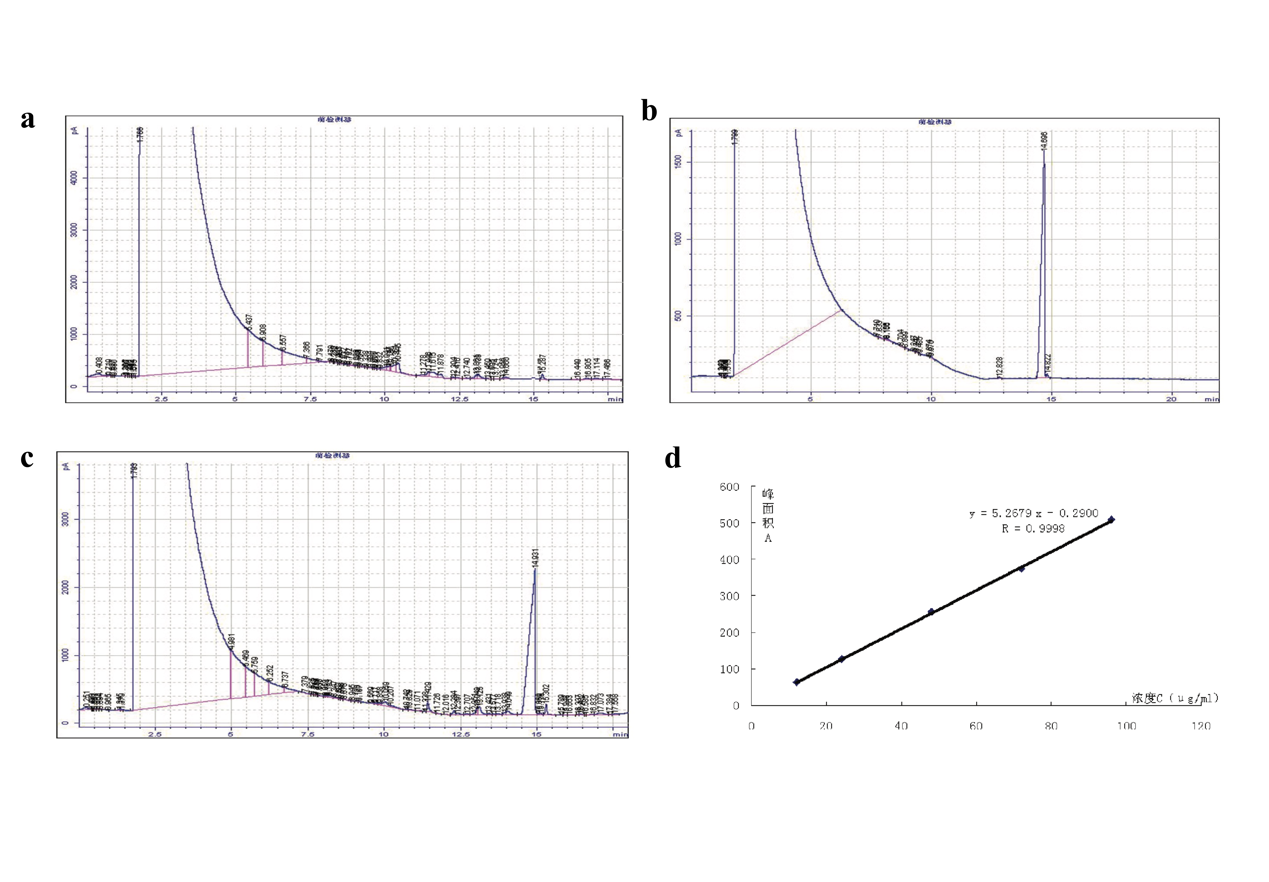


**Fig.2. Muscone content in NQ**

**(a)**The **gas chromatography (**GC)of negative mode. **(b)**The GC of the muscone reference substance. **(c)** The GC of NQ. **(d)** Standard curve of muscone.

## Materials and methods

**1.2.1 Metabolite extraction**


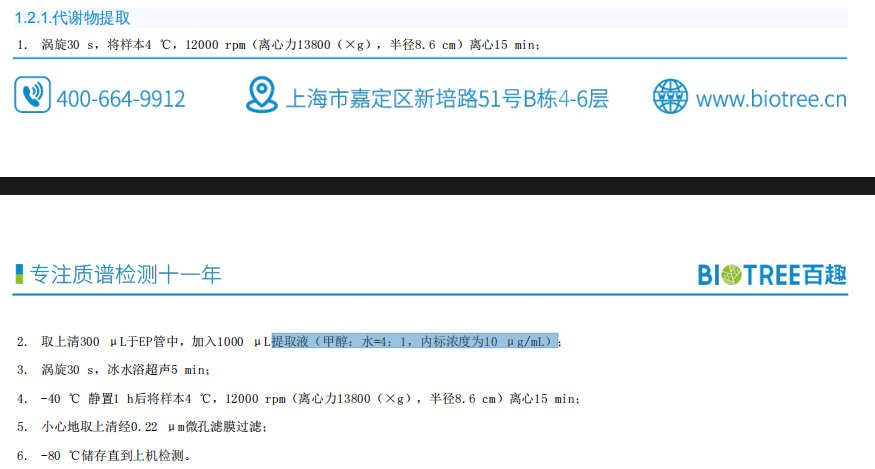

1. vortex for 30 s, centrifuge the sample at 4 C, 12000 rpm (centrifugation force 13800 (Xg), radius 8.6 cm) for 15 min.

2. take 300 L of supernatant in an EP tube, add 1000 L of extraction solution **(methanol: water = 4:1, internal standard concentration of 10 ug/mL**).

3. vortex for 30 s, sonicate the sample on an ice-water bath for 5 min.

4. -40 CC stand for 1 h After that, the samples were centrifuged at 4 C for 15 min at 12000 rpm (centrifugal force 13800 (Xg), radius 8.6 cm).

5. The supernatant was carefully filtered through 0.22 wm microporous filter membrane.

6. The samples were stored at -80 C until assayed on the machine.

**1.2.2 Ultrahigh performance liquid chromatography quadrupole exactive orbitrap mass spectrometry (UHPLC-QE-MS)** **technique for the determination of Chinese medicine compound (botanicals) with the following detailed parameters**


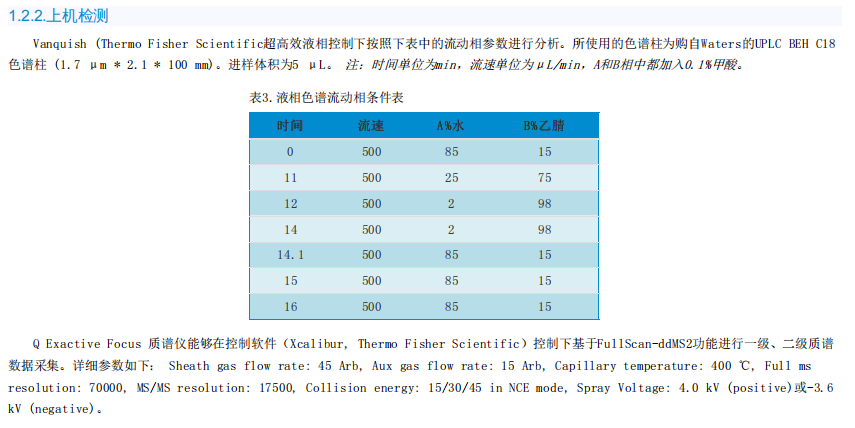


Vanquish (Thermo Fisher Scientific UHPLC) was analysed according to the mobile phase parameters in the table below. The column used was a UPLC BEH C18 column (1.7 um * 2.1*100 mm) from Waters. The injection volume was 5 WL. Note: The time is in min, the flow rate is in M L/min, and 0.1% formic acid was added to both phase A and B. The Q Exactive Focus mass spectrometer was able to perform primary based on the FullScan-ddMS2 function under the control software (Xcalibur, Thermo Fisher Scientific), The Q Exactive Focus mass spectrometer is capable of primary and secondary mass spectrometry data acquisition based on the FullScan-ddMS2 function under the control software (Xcalibur, Thermo Fisher Scientific). The detailed parameters are as follows: Sheath gas flow rate: 45 Arb, Aux gas flow rate: 15 Arb, Capillary temperature: 400 C, Full ms resolution: 70000, MS/MS resolution: 17500, Collision energy: 15/30/45 in NCE mode, Spray Voltage: 4.0 kV (positive) ak-3. 6 kV (negative).

**1.2.3** **Data processing**

After the raw data were converted into mzXML format by ProteoWizard software, the metabolite identification was performed using the collaboratively written R package, the databases used were Biotree TCM (V 1.0) and BT-HERB (V 1.0), and then visualised and analysed using the independently written R package.The criteria for substance identification are shown below:


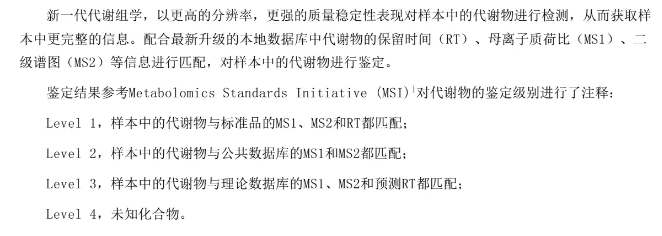


The new generation of metabolomics, with higher resolution and stronger quality stability performance to detect metabolites in the sample, so as to obtain more complete information in the sample. The metabolites are identified by matching the retention time (RT), parent ion mass-to-charge ratio (MS1), and secondary spectra (MS2) of the metabolites in the newly upgraded local database.

The metabolite identification levels are annotated with reference to the Metabolomics Standards Initiative (MSI):

Level 1, the metabolite in the sample matches the MS1, MS2 and RT of the standard;

Level 2, where the metabolite in the sample matches both MS1 and MS2 in the public database:

Level 3, metabolites in the sample matched both MS1, MS2 and predicted RT from the theoretical database;

Level 4, unknown compounds.

It can also provide information on MZ, RT, and secondary ion fragments (MS2) in the stereotype table, as shown below.


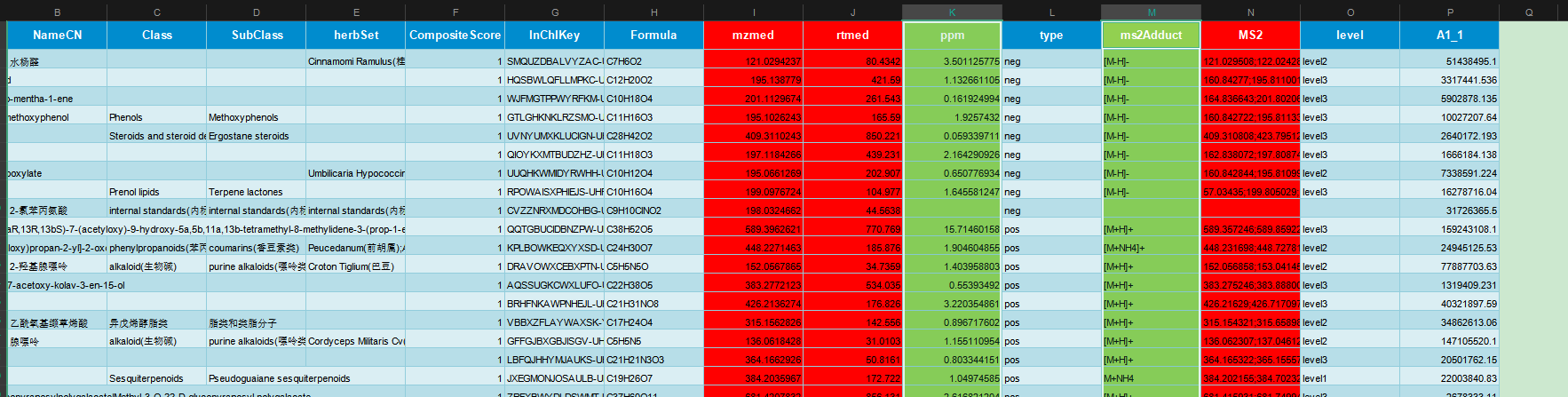

Supplement: Supplementary file 3 [file DataSheet1.docx]
